# Treatment of critically ill children with kidney injury by sustained low-efficiency daily diafiltration

**DOI:** 10.1007/s00467-012-2254-1

**Published:** 2012-08-18

**Authors:** Chia-Ying Lee, Huang-Chieh Yeh, Ching-Yuang Lin

**Affiliations:** 1 Division of Pediatric Nephrology, Children’s Medical Center, China Medical University Hospital, China Medical University, Taichung, Taiwan; 2 Division of Nephrology, Department of Medicine, China Medical University and Hospital, Taichung, Taiwan; 3 College of Medicine, China Medical University, No.2, Yuh-Der Rd., Taichung, 40402 Taiwan; 4 Clinical Immunology Center, China Medical University Hospital, No.2, Yuh-Der Road, Taichung, 40402 Taiwan

**Keywords:** Acute kidney injury, Continuous renal replacement therapy, Intermittent hemodialysis, Sustained low efficiency daily diafiltration, Critically ill children

## Abstract

**Background:**

Continuous renal replacement therapy (CRRT) and intermittent hemodialysis (IHD) offer diverse benefits and drawbacks for critically ill children with acute kidney injury (AKI). Sustained low-efficiency daily diafiltration (SLEDD-f) involves a conceptual and technical hybrid of CRRT and IHD. We report our SLEDD-f application to critically ill children in the pediatric intensive care unit (PICU).

**Methods:**

SLEDD-f was delivered by the new Fresenius 5008 therapy system with blood flow 5 ml/kg/min, dialysate flow 260 ml/min, hemofiltration 35 ml/kg/h for 8–10 h daily. Changes in blood pressure, blood gas, electrolyte, hemoglobulin (Hb), and hematocrit (Hct) were closely monitored.

**Results:**

From February 2010 to June 2011, 14 critical patients with a total of 60 SLEDD-f sessions were studied retrospectively. Heparin was used in 46 sessions (76.6%) with no bleeding complications. Hypertension above 135 mmHg returned to normal, hypotension below 90 mmHg showed no drop. Metabolic acidosis and hyperkalemia normalized. Elevated Hb, Hct, and their ratio revealed improving hemodilution. Three episodes of intradialytic hypotension (5.0%) and one of circuit clotting (1.7%) led to premature termination. The 28-day survival rate was 71.4%.

**Conclusions:**

This pilot investigation demonstrates that SLEDD-f provides good hemodynamic tolerance and correction of fluid overload, pH, and electrolyte imbalance for critically ill children with AKI.

## Introduction

Two classic options for acute kidney injury (AKI) are intermittent hemodialysis (IHD) and continuous renal replacement therapies (CRRT), each with benefits and drawbacks. IHD is applied with the diffusion principle and performed for around 4 h per session every other day. CRRT with convection only or combined diffusion is performed nonstop. The superior modality for AKI in adults remains controversial [1], but CRRT has seen widest use in children with critical AKI because of continuous programmed fluid removal plus better hemodynamic tolerance [2–4]. Yet CRRT frequently gives rise to complications in such cases: e.g. intradialytic hypotension, hemorrhage, electrolyte disturbance [5]. The ideal renal replacement for children should provide adequate hemodynamic stability, good volume control, solute removal, high efficacy in correcting acid-base abnormalities, and electrolyte imbalance. It should involve minimal work with limited cost, be nonlabor-intensive and/or technically demanding, provide dialysis-free time out of the pediatric intensive care unit (PICU) for diagnostic and therapeutic procedures while enhancing renal recovery and survival without complications [1, 6]. IHD is not well tolerated owing to rapid fluid shift and poor quality of life during therapy. CRRT is better tolerated, yet complications and cost-effectiveness pose problems, especially in resource-limited nations [4, 5]. Sustained low-efficiency daily diafiltration (SLEDD-f), a conceptual and technical hybrid of IHD and CRRT, is an increasingly popular method of renal replacement for AKI patients [1, 7]. Adult studies have yielded excellent clinical and metabolic outcomes in critically ill AKI patients [7, 8]. To our knowledge, no one has ever reported on SLEDD-f usage in children; we thought it worthwhile to share our experience and rate the clinical efficacy, quality of life, and cost analysis of SLEDD-f therapy in critically ill children with AKI.

## Materials and methods

### Patients

The progress records of 14 critical AKI patients from February 2010 to June 2011 with a total of 60 SLEDD-f sessions in PICU who met the criteria for RRT were retrospectively collected and reviewed. Children with body weight (BW) less than 20 kg, brain death or severe neurological involvement were excluded. Because of a lack of proper hemodiafilters and tubing sets for SLEDD-f in children less than 20 kg in Taiwan, we preferred continuous veno-venous hemofiltration (CVVH) for them, giving SLEDD-f top priority to larger children and adolescents. For brain death and severe neurological involvement, palliative care is considered first. We respect family opinions and suggestions from pediatric neurologists, detailing the advantages and disadvantages of RRT to them.

Data and clinical information included demographic characteristics (age, gender, and body weight), diagnosis, Pediatric Risk of Mortality III (PRISM III) score, percentage of fluid overload (%FO), and outcome. Decisions on SLEDD-f were reached by both the attending intensivist and the nephrologist according to indications, such as elevated blood urea nitrogen (BUN) and serum creatinine (sCr), oliguria, acute volume overload or electrolyte imbalance refractory to other therapy. According to ambulatory blood pressure monitoring (ABPM) and reference values for ABPM in normal children and adolescents in Taiwan [9], we defined systolic blood pressure (sBP) above 135 mmHg as hypertension and below 90 mmHg as hypotension. Definitions of AKI and end-stage renal disease (ESRD) are based on pediatric-modified RIFLE criteria [10]. Multi-organ dysfunction syndrome (MODS) meant that the underlying primary disease leading to AKI affected at least one organ system other than the kidneys [11]. The percentage of fluid overload (%FO) was calculated as ${{{\left( {{\text{fluid}}\;{\text{in}} - {\text{fluid}}\;{\text{out}}} \right)}} \left/ {{\left( {{\text{ICU}}\;{\text{admission}}\;{\text{weight}}} \right)}} \right.} \times {1}00\%$ [12]. Technical information regarding SLEDD-f prescription included blood flow rate, dialysate flow rate, real duration, replacement fluid, ultrafiltration volume, heparin dose and complications. Vital signs, blood gas, electrolyte, and biochemistry data were closely monitored before, during, and after SLEDD-f therapy.

To evaluate the changes in inflammatory markers, we collected serum before and after SLEDD-f. Levels of adiponectin, IL-17A, and IL-6 were measured in duplicate by ELISA kits (Assay-Pro Inc., Brooklyn, NY, USA), as per the manufacturer’s instructions.

A questionnaire was specifically designed for ESRD [13], dialysis [14], and modified quality of life evaluation [15, 16] and was applied in Taiwan with fair reliability (Cronbach’s α between approximately 0.888 and 0.924) and validity (Pearson correlation coefficient between approximately 0.279 and 0.669). We chose the most common complaints from AKI patients and evaluated their change before and after SLEDD-f. Chen and Ku [16] found physical symptom distress and quality of life negatively correlated. Among 25 items of physical distress, we chose subjective symptoms and perceptions: appetite, sleep, static activity limitation, chest pain, palpitation, and dyspnea. The first three items had 1–5 score, and the last three symptoms 1–3, according to the degree of symptom improvement (a higher score signifying greater improvement). Children had to be conscious and able to answer simple questions. Rating was based on medical and nursing records.

### Components of SLEDD-f

Treatment was performed with a Fresenius 5008 on-line hemodiafiltration system. Polysulfone high flux hemodiafilter, FX60 (membrane surface 1.4 m^2^ and priming volume 74 ml) and FX40 (membrane surface 0.6 m^2^ and priming volume 32 ml) were used for BW above and below 40 kg respectively. For operating parameters, blood flow rates were set at 5 ml/kg/min for BW 20–40 kg or 200 ml/min for BW above 40 kg, countercurrent dialysate flow to 260 ml/min and filtration rate to 35 ml/kg/h in pre-dilution mode. According to blood flow (Qb) and filtration rate (Qf), total pre-dilution replacement fluid was calculated as $\left[ {{{{\left( {{\text{Qf}} \times {\text{Qb}}} \right)}} \left/ {{\left( {{\text{Qb}} - {\text{Qf}}} \right)}} \right.}} \right] \times {6}0 \times {\text{treatment}}\;{\text{duration}}\;\left( {\text{hours}} \right)$. Treatment lasted 8–10 h daily. Constituent concentration of dialysate and replacement fluid were: [Na^+^]:138 mEq/L, [K^+^]:2.0 mEq/l, [Cl^-^]:109.5 mEq/l, [Mg^2+^]:1.0 mEq/l, [Ca^2+^]: 3.0 mEq/l, [HCO_3_^-^]:32 mEq/l. Anticoagulation infusion in the extracorporeal blood circuit was unfractionated heparin with a loading dose of 10–20 IU/kg and maintenance dose 5–10 IU/kg/h. For those with prolonged activated partial thromboplastin time (aPTT) >75 s, international normalized ratio (INR) >2, activated clotting time (ACT) >275 s, platelet count <50,000/μl, and/or clinical presentation or suspect bleeding diathesis, we lowered the heparin dose or held it steady. In most cases, we used aPTT as the parameter and adjusted the heparin dose. We checked aPTT at 0, 3, and 6 h from the arterial site: if aPTT <35 s or below baseline, heparin 500 IU bolus and add 200 IU/h; if sPTT >35 s, but <45 s, add heparin 200 IU/h; if aPTT >45 s but <55 s, add heparin 100 IU/h; if aPTT >55 s, but <65 s, keep heparin at maintenance dose; if aPTT >65 s but <75 s, subtract heparin 50 IU/h; if aPTT >75 s, hold heparin. If any of these parameters (aPTT, INR, ACT, platelet count) or clinical presentation of bleeding existed, such as gastric ulcer with bleeding or epistaxis, we held heparin or adjusted it to the lowest dose. The machine is able to auto-flush normal saline each hour to prevent tube clotting.

### Statistics

All values in figures and tables are expressed as mean ± standard error. Student’s *t* tests (unpaired, two-tailed) were used for intergroup comparison, with *p* value <0.05 indicating statistical significance. Analysis was performed with SPSS/Windows software.

## Results

### Patient characteristics

From February 2010 to June 2011, 14 patients received a total of 60 SLEDD-f treatments. Mean age was 14.9 ± 2.3 years, mean body weight was 54.4 ± 23.3 kg. Average PRISM III scores were 16.8 ± 23.3. Five patients had PRISM III scores under 10, another 9 over 10. Table 1 details the clinical characteristics.

**Table 1:** Clinical characteristics in patients receiving sustained low-efficiency daily diafiltration (SLEDD-f)

Number	Diagnosis	PRISM score	Number of treatments	Number of pressors	AKI or ESRD	Pulmonary edema	MODS	28-day survival
1	Methylmalonic acidemia, renal and liver transplantation, shock	30	4	3	AKI	Yes	Yes	No
2	Mediastinal yolk sac tumor, cardiogenic shock	22	2	2	AKI	Yes	Yes	Yes
3	Lymphoma, stem cell transplantation, GVHD, shock	26	2	3	AKI	Yes	Yes	No
4	ESRD, renal transplantation, recurrent FSGS	11	3	0	ESRD	Yes	No	Yes
5	ESRD, intolerance to IHD	9	2	0	ESRD	Yes	No	Yes
6	SLE, lupus nephritis, refractory nephrotic syndrome, intolerance to IHD	9	11	0	AKI	Yes	No	Yes
7	SLE, lupus nephritis, refractory nephrotic syndrome, *Klebsiella pneumonia* septic shock	35	3	3	AKI	Yes	Yes	No
8	Diabetic ketoacidosis	10	4	0	AKI	Yes	No	Yes
9	ESRD, renal transplantation, chronic rejection, septic shock	24	4	3	AKI	Yes	Yes	No
10	SLE, lupus nephritis, influenza A infection with acute respiratory distress syndrome	31	7	1	AKI	Yes	Yes	Yes
11	SLE, lupus nephritis, refractory nephrotic syndrome	4	3	0	AKI	Yes	No	Yes
12	Crescentic glomerulonephritis IgA nephropathy	7	8	0	AKI	Yes	No	Yes
13	Crescentic glomerulonephritis	7	3	0	AKI	Yes	No	Yes
14	FSGS progressing to ESRD, cachexia	10	4	0	ESRD	No	No	Yes

AKI: acute kidney injury; ESRD: end-stage renal disease; FSGS: focal segmental glomerulosclerosis; MODS: multi-organ dysfunction syndrome; GVHD: graft-versus-host disease; SLE: systemic lupus erythematosus; PRISM: pediatric risk of mortality; IHD: intermitent hemodialysis

Of the 3 ESRD patients, 1 girl with renal transplantation returned to dialysis owing to recurrent focal segmental glomerulosclerosis (FSGS) with progressive pulmonary edema and intolerance to IHD; 1 girl suffered from progressive generalized edema, hypertension and intolerance to IHD; and 1 boy with progressive FSGS to ESRD, cachexia, and intolerance to IHD, but the peritoneal dialysis tube was not implanted. Intolerance to IHD and poor ultrafiltration were indicators in these 3 ESRD patients.

Eleven patients had AKI: 2 with renal transplant had multi-organ dysfunction syndrome (MODS); 1 had a stem cell transplant with graft-versus-host disease (GVHD) and MODS; 1 with diabetic ketoacidosis and pulmonary edema; 2 with crescentic glomerulonephritis showed renal damage progression; 1 with a mediastinal yolk sac tumor suffered from cardiogenic shock, respiratory failure, and MODS; 1 had lupus nephritis superimposed influenza A infection complicated by acute respiratory distress syndrome (ARDS) and MODS; 2 girls with lupus nephritis had refractory nephrotic syndrome with pulmonary edema; another girl with lupus nephritis suffered from refractory nephrotic syndrome and septic shock with *Klebsiella pneumonia* complicated with MODS.

Thirteen patients had pulmonary edema; 5 had respiratory failure and needed positive airway pressure or intubation and mechanical ventilation. Six patients diagnosed with MODS all needed inotropic agents: 4 received three kinds of inotropic agents and died within 4 weeks.

For 7 critical patients with relative hemodynamic stability, sessions of IHD were performed; premature termination resulted in poor ultrafiltration due to chest tightness, angina, dizziness, tachycardia, and hypotension. They tolerated SLEDD-f well, with adequate ultrafiltration, subsiding pulmonary edema, and few complications. Quality of life improvements included better sleep, appetite, and static activity, all without discomfort.

### Improvement in blood pressure control

**Fig. 1:**
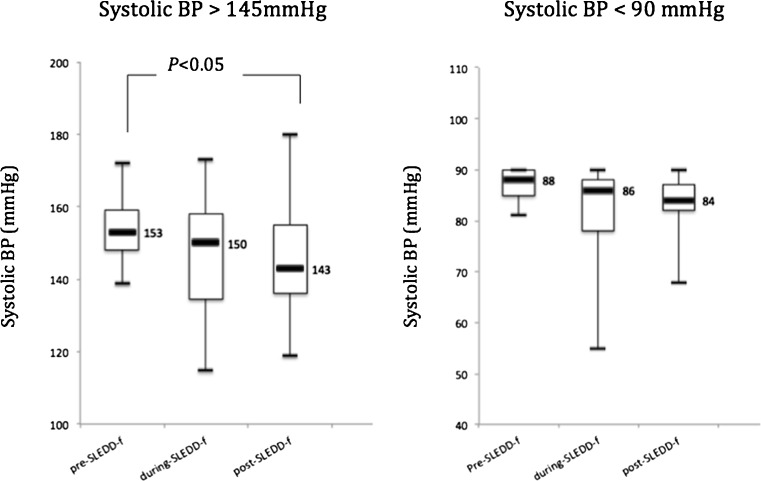
Changes in blood pressure (BP) in hypertensive (systolic BP > 145 mmHg) and hypotensive (systolic BP < 90 mmHg) patients before, during, and after sustained low-efficiency daily diafiltration (SLEDD-f) therapy.* Boxes* and* bars* represent the interquartile range and the median value respectively. The middle number represents the median value. In a hypotensive group, the comparison did not reach statistical significance

Average treatment duration was 8.0 ± 0.5 h and fluid removal per treatment was 2,390 ± 103 ml. In patients with hypertension, mean sBP 153.5 ± 9.4 mmHg before SLEDD-f fell to 145.3 ± 17.2 mmHg (*p* < 0.02) after treatment; on the other hand, for patients with hypotension, a mean sBP 87.0 ± 3.5 mmHg before SLEDD-f became 80.9 ± 11.4 mmHg during treatment (*p* = 0.15; Fig. 1), revealing insignificant depression. Three episodes of intradialytic hypotension (3 out of 60, 5.0%) led to premature termination. There was no apparent increase in dose or de novo institution of inotropic agents during and after treatment.

### Improvement of pulmonary edema and pulmonary hemorrhage

**Fig. 2:**
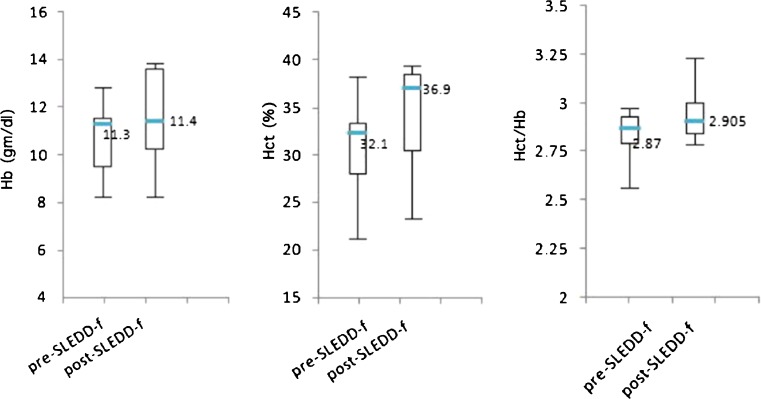
Changes in hemodilution before and after sustained low-efficiency daily diafiltration (SLEDD-f) therapy. *Boxes* and *bars* represent interquartile range and median value respectively. The middle number represents the median value. None of these comparisons reached statistical significance

**Fig. 3:**
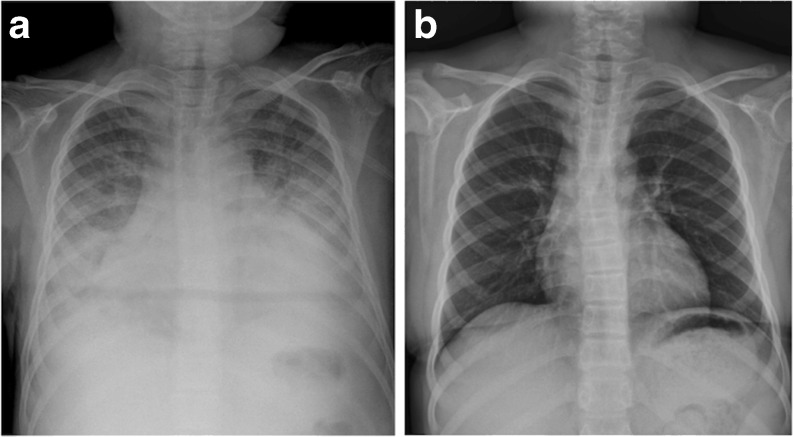
Changes in pulmonary edema before and after sustained low-efficiency daily diafiltration (SLEDD-f) therapy. **a** Significant lung marking, blunting costophrenic angle, and cardiomegaly before SLEDD-f. **b** Condition improved after SLEDD-f therapy

To evaluate correction of hemodilution, we recorded changes in hemoglobin (Hb), hematocrit (Hct), and their ratio (Hct/Hb). Before SLEDD-f, mean Hb, Hct, and Hct/Hb ratio were 10.6 ± 1.6 g/dl, 30.5 ± 5.4%, and 2.83 ± 0.15. SLEDD-f changed these data to 11.6 ± 2.2 g/dl, 33.8 ± 6.0% and 2.95 ± 0.16 respectively (Fig. 2). A potential trend existed towards improvement (although not statistically significant), with no apparent intradialytic hemorrhage or blood consumption. Pulmonary edema (Fig. 3) improved after SLEDD-f therapy. A 14-year-old girl with active SLE and refractory nephrotic syndrome suffered from sudden onset of cyanosis, dyspnea, and hematemesis. Chest X-ray revealed a diffuse alveolar pattern, implying alveolar hemorrhage. SLEDD-f therapy and aggressive immunosuppressant treatment resulted in subsidence of pulmonary hemorrhage, and the ventilation and oxygenation improved gradually.

### Improvement of hyperkalemia and metabolic acidosis

**Fig. 4:**
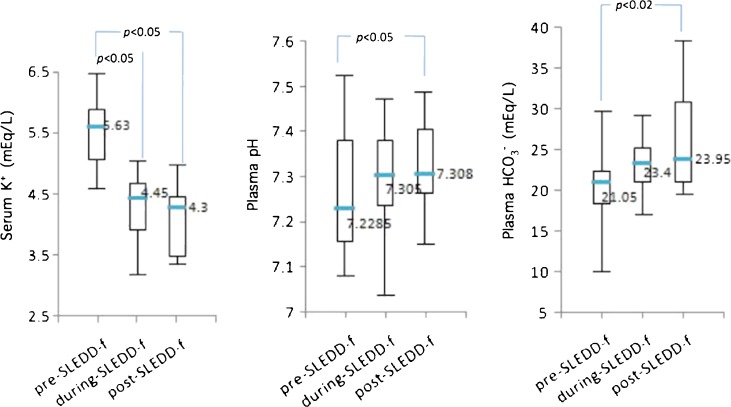
Changes in serum potassium and metabolic acidosis before and after sustained low-efficiency daily diafiltration (SLEDD-f) therapy.* Boxes* and* bars* represent interquartile range and the median value respectively. The middle number represents the median value. Differences between pre- and post-SLEDD-f therapy were statistically significant

Hyperkalemia appeared in 5 patients (mean serum potassium 5.6 ± 0.8 mEq/l before SLEDD-f) and was corrected to within the normal range (4.3 ± 0.7 mEq/l during and 4.1 ± 0.7 mEq/l after SLEDD-f, *p* < 0.05). Those with metabolic acidosis regained normal plasma levels (7.27 ± 0.16 pre-SLEDD-f and 7.32 ± 0.11 post-SLEDD-f, *p* < 0.05), low HCO_3_ returning to within a normal range (20.3 ± 5.7 and 26.5 ± 7.0 mEq/l respectively, *p* < 0.02; Fig. 4). Sodium, calcium, magnesium and/or phosphate showed no significant change.

### Decreased inflammatory markers

We checked the serum inflammatory markers adiponectin, interleukin 17A (IL-17A), and IL-6 from only 8 survivors. Before SLEDD-f, serum adiponectin levels were 3,835.0 ± 1,011.7 pg/ml, IL-17A levels 1,248.2 ± 212.6 pg/ml and IL-6 levels 204.5 ± 100.6 pg/ml. After SLEDD-f, serum adiponectin levels fell significantly to 661.3 ± 377.5 pg/ml (*p* < 0.001), IL-17A to 10.4 ± 7.6 pg/ml (*p* < 0.001) and IL-6 levels to 61.9 ± 25.7 pg/ml (*p* < 0.001).

### Complication during SLEDD-f

Heparin was prescribed in 46 out of 60 treatments (76.6%), without bleeding complications. In 14 treatments without heparin, one episode of circuit clotting (1/60, 1.7%) occurred and led to premature termination.

### Mortality

Four patients died; 28-day survival was 71.4%. According to the PRISM III score, 5 patients with PRISM III score <10 had 100% survival; 9 with PRISM III score >10 had 55.6% survival. Four patients received renal (3 patients) or stem cell (1 patient) transplantation, and 3 died. A 17-year-old boy with methylmalonic acidemia (MMA) after liver and renal transplantation suffered from severe hemorrhagic peptic ulcer with hypovolemic shock, systemic cytomegalovirus disease,* Pneumocystis jiroveci* pneumonia with respiratory failure and hepatorenal syndrome. A 13-year-old girl with FSGS progressing to ESRD after a renal transplant had an acute rejection, pneumonia with respiratory failure and septic shock. The other 17-year-old boy with lymphoma after stem cell transplantation suffered from graft-versus-host disease (GVHD), AKI, respiratory failure, and liver failure. One girl with renal transplantation survived, but returned to ESRD because of recurrent FSGS and acute rejection. A 14-year-old girl with SLE had refractory nephrotic syndrome and died of *Klebsiella pneumonia*-related septic shock. These 4 had MODS (Table 1).

Comparison of survivors and nonsurvivors (Table 2) shows similar age, BW, eGFR, and BUN before SLEDD-f. Nonsurvivors had a significantly higher PRISM III score, %FO, MODS, and vasopressor usage. Of 4 nonsurvivors, 1 boy received bone marrow transplantation, another had a concurrent liver and kidney transplant, while another had a renal transplant. All had high PRISM III scores, clinical fluid overload, and MODS.

**Table 2:** Patients’ characteristics by survival status

Variable	All	Survival	Non-survival	*p v*alue
	*n*= 14	*n*= 10	*n* = 4	
Age (years)	14.9 ± 2.3	14.7 ± 2.5	15 ± 2.4	0.426
Weight (kg)	54.4 ± 23.3	57.1 ± 25.9	55.8 ± 17.2	0.463
PRISM at SLEDD-f	16.8 ± 10.6	12 ± 8.2	28.8 ± 4.9	0.0015
%FO before SLEDD-f	4.7 ± 3.0	3.8 ± 2.9	7.1 ± 1.8	0.023
Inotropes (number at SLEDD-f)	1.1 ± 1.4	0.3 ± 0.7	3	<0.001
MODS	42.9%	20%	100%	<0.001
eGFR before SLEDD-f	35.2 ± 32.5	33.3 ± 31.6	39.8 ± 39.5	0.375
BUN before SLEDD-f	93.9 ± 66.6	88.2 ± 74.2	108.2 ± 48.2	0.315
Transplantation	4	1	3	0.0045
Shock	4	1	3	<0.001

%FO: fluid overload %; PRISM: Pediatric Risk of Mortality; SLEDD-f: Sustained low-efficiency daily diafiltration; MODS: multi-organ dysfunction syndrome; eGFR: estimated glomerular filtration rate given in ml/min/1.73 m2; BUN: blood urea nitrogen, mg/dl. *P* < 0.05 represents a significant association between surviving and nonsurviving patients

### Analysis of quality of life

**Fig. 5:**
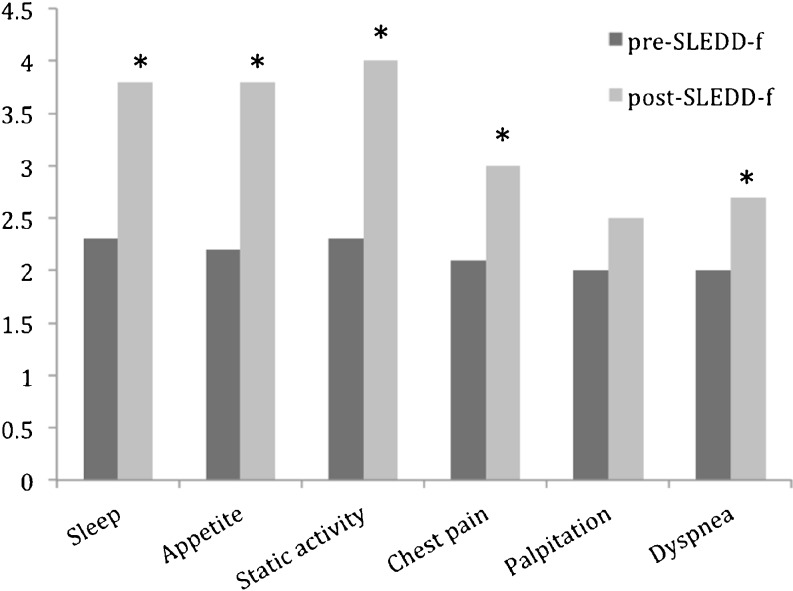
Changes in quality of life before and after sustained low-efficiency daily diafiltration (SLEDD-f) therapy.* Asterisk* represents *p* < 0.05

In 34 SLEDD-f trials for 6 patients, quality of life analyses were performed. According to medical charts and nursing records, sleep quality, appetite, static activity, and objective symptoms (chest pain or tightness, palpitation, and dyspnea) scores improved after SLEDD-f (Fig. 5).

### Cost analysis

Hemodiafilter and tubing for CVVH costs US$235, versus about US$69 for SLEDD-f. Because of the longer duration, CVVH needs larger amounts and more expensive replacement fluid (US$70) than the dialysate needed for SLEDD-f (US$8). The average cost of each SLEDD-f treatment was US$77 per day, as opposed to CRRT with an average of US$305. It is hard to calculate personnel fees under a different nation’s insurance system. Given limited resources, it is important to select treatments with greater or equal efficacy and cheaper consumable materials.

## Discussion

The present study is the first report of SLEDD-f treatment to analyze hemodynamic stability, volume control, acid-base, electrolyte correction, quality of life, and survival in critical children. SLEDD-f proves to be less costly than CVVH in the realm of consumable materials. Patients intolerant to IHD, while treated with SLEDD-f, enjoyed better efficacy, more stable hemodynamics and better quality of life. Further comparison will be needed to demonstrate these advantages.

In adults, controversy exists over superior survival after IHD or CRRT [17]. Differences in clinic, technique, and distribution of extracellular and intracellular fluid between populations make it unwise to apply CRRT adult data to children. Recent studies [2, 18, 19] have suggested CRRT to be the preferred modality for critical children. Appropriate CRRT merits special consideration, given the range of body weight and composition of intra- and extracellular fluid. Owing to immature renal tubular function, metabolic acidosis and electrolyte disturbance develop more readily in children than in adults. During CRRT delivery, a substantial proportion of blood volume was pumped through the extracorporeal circuit. Children developed more complications with CRRT. It is difficult to establish catheterization with relatively larger caliber central venous or double lumen catheters; more accurate fluid control is needed, since the larger extracorporeal volume of the circuit may predispose to hypotension. In lower body weight patients, extracorporeal blood volume exceeds 10% of blood volume, exposing them to risks associated with hemodynamic instability [4] and adverse transfusion reaction: anaphylaxis, hemolysis, infection, lung injury, and GVHD [20].

Two major studies on pediatric CRRT [21, 22] revealed diffusion-based continuous veno-venous hemodiafiltration (CVVHDF) and hemodialysis (CVVHD) to be the favored modalities (74% and 78%). Better survival was noted among patients treated with CVVHD and CVVHDF than among those treated with CVVH [21]. Marshall et al. [7] showed that SLEDD-f afforded a greater dose of dialysis than IHD and could provide small solute clearance comparable to that from a CRRT regimen with substitution fluid rate of 35 ml/kg/h. Based on the theoretical data presented, increases in either Qf or treatment duration could be easily induced as part of routine SLEDD-f to afford a similar amount of convective clearance as CRRT. SLEDD-f is expected to have better patient outcome and wider clinical application.

Acute kidney injury is no isolated event, and it engenders distant organ injury to lungs, heart, liver, and brain via a mechanism involving neutrophil migration, higher cytokine concentration, and oxidative stress [23]. Understanding the mechanism behind AKI-induced distal organ injury is vital, as it may reveal new therapeutic targets. We found a significant decrease in serum adiponectin, IL-17A, and IL-6 level in survivors after SLEDD-f, a phenomenon that might represent an association between removing certain cytokines and AKI outcome, but this study is limited in terms of its small number and retrospective design.

Adiponectin, an adipokine, is produced almost exclusively by adipocytes and circulates in high concentrations in human plasma [24, 25]. Alteration in blood adiponectin concentrations has been linked to many human diseases in numerous cross-sectional and prospective studies. Adiponectin is markedly increased in patients with nephrotic syndrome; proteinuria is strongly related to circulating adiponectin in patients with nephrotic syndrome and non-nephrotic renal disease [26]. Investigation of critical illness [25] revealed an inverse correlation between adiponectin and inflammatory cytokines; whether this emanates from the disease process itself, or whether patients with lower levels of this hormone are more susceptible to critical illness is unknown. However, there were no data on the association among adiponection, AKI, diafiltration, and prognosis. Our study revealed decreased adiponectin levels after SLEDD-f in 8 survivors. The phenomenon might imply either SLEDD-f removal of adiponectin or represent a natural recovery from a critical condition. This limited retrospective study had neither control nor comparative groups; in future we aim to conduct better-designed research to investigate these possibilities.

IL-17A is a proinflammatory cytokine released by both T cells and innate immune cells, and plays a key role in both innate and adaptive immunity [27]. Convincing experimental evidence indicates that IL-17-producing T cells contribute to kidney injury in renal inflammatory diseases [28, 29]. In animal studies after ischemic or non-ischemic AKI, plasma TNF-α, IL-17A, and IL-6 increased sharply, leading to small intestine and liver injury [30]. In critically ill patients, controversial results revealed that IL-17 might induce multiple organ dysfunctions [31–33]. In our study, high serum IL-17A decreased significantly after successful SLEDD-f treatment in 8 survivors. Changes in serum adiponectin, IL-17A, and IL-6 might indicate that SLEDD-f is able to remove certain inflammatory markers. However, incomplete data on the clearance of urea and creatinine limited the possibility of rating the efficacy of SLEDD-f in terms of depuration. Further research is needed in this area.

Pulmonary edema, acid-base disturbance and/or electrolyte imbalance were principal indications for renal replacement therapy. IHD is the most efficient modality for fluid, metabolic, and electrolyte control. Yet rapid fluid shifts are not well tolerated in complicated and unstable clinical conditions [4]. Such patients better tolerate CRRT hemodynamically; hemorrhage and/or electrolyte disturbance represent major complications [5]. Our 3 pulmonary edema patients who were intolerant to IHD adapted to SLEDD-f well, showing improved symptoms and quality of life.

Children tend to bleed more than adults [34, 35] during maturation of the hemostatic system. According to the literature regarding the complications of CRRT in critically ill children [5], 10% on CRRT presented clinically significant hemorrhage, with no correlation between the presence of hemorrhage and age, weight, diagnosis or clinical severity at the start of CRRT. Though differences did not reach statistical significance, they indicated higher mortality in patients with rather than without hemorrhage. Heparin use requires careful adjustment to avoid either hemorrhage or circuit clotting. Hemorrhage in critically ill children with CRRT could arise from a number of factors: coagulation disorder, platelet dysfunction, altered tissue perfusion, CRRT itself, and anticoagulation. When our SLEDD-f patients had any clinical presentation of bleeding or suspected hemorrhagic diathesis, heparin was adjusted or held. No such bleeding complication appeared in our patients, but one episode of circuit clotting occurred at 3.5 h and led to premature termination in a patient with multi-organ involvement.

Hypotension is a key problem for critically ill children. Hypotension at CRRT connection is one of the most common CRRT-related complications [5]. After smooth connection, CRRT is advantageous for hemodynamically unstable patients as it need not be terminated because of hypotension, unlike IHD. All our patients had smooth connection, while three episodes of intra-dialytic hypotension occurred in shock cases. We tried our best to slow down blood flow, raise the inotropic dose, and cease ultrafiltration, yet slower blood flow, disseminated intravascular coagulation (DIC), and thrombosis resulted in tube clotting and discontinuation of therapy. The need to terminate therapy depended on RRT modality effects as well as clinical conditions. This is also common for CVVH, especially for unstable hemodynamic patients, in our experience. The small number of patients precluded us from determining the superiority of either SLEDD-f or CVVH.

Mortality in critically ill children requiring CRRT is reported to be between 35.6 and 57.0% [21, 22, 36–38]. Different diseases and demographic traits correlate with diverse mortality rates. In adults on CRRT, risk factors associated with mortality were age, greater clinical severity score, hemodynamics, sepsis, and respiratory failure. In a prior study by Symons et al., mortality rose with lower body weight (<10 kg), younger age (<1 year), higher PRISM II score (>10), combined fluid overload, metabolic acidosis, and multi-organ involvement [22]. In the prospective pediatric continuous renal replacement therapy (ppCRRT) registry group, survival of patients with PRISM II score <10 was 55%; for those with >10, 47% [22]. In our report of those treated with SLEDD-f, the percentages were 100% and 55.6% in patients with PRISM III score <10 and >10 respectively. Besides PRISM III score, other parameters, such as pre-RRT multiple organ failure, fluid overload, hemodynamic disturbance, and administration of cardioactive drugs can offer more reliable prognostic indicators. Further analysis of critically ill children with MODS receiving CRRT in the ppCRRT registry group [11] highlighted the PRISM score, central venous pressure (CVP), and the percentage of fluid overload (%FO) at CRRT initiation as being sharply lower for survivors versus nonsurvivors. Table 2 compares surviving and nonsurviving patients: nonsurvivors more frequently suffered from MODS and had higher PRISM scores, %FO at ICU, and inotropic agent usage before SLEDD-f initiation. Patients requiring SLEDD-f for primary or predominant renal disease tallied lower PRISM scores and had 100% survival. Early SLEDD-f intervention before severity progresses and fluid overload increases may improve the patient survival rate and decrease morbidity. In ppCRRT [22], the survival rate in liver disease and/or transplant patients was lower than the overall rates. Among nonsurviving patients, 2 with renal and 1 with stem cell transplant suffered from shock and MODS, including respiratory failure, AKI, and liver failure. Transplantation and shock predisposed to a poor prognosis.

This report is limited by its retrospective design, small patient numbers, lack of control groups for diverse treatments, and the fact that there were no young patients weighing less than 20 kg, who are usually reported to be at a higher risk of complications and death. With respect to quality of life, a further limitation was the fact that it was based on a retrospective review of medical and nursing records, not an ad-hoc questionnaire.

In conclusion, our pilot study showed that the advantages of SLEDD-f for critically ill children with renal injury included good hemodynamic tolerance, stable BP, well controlled fluid overload, good correction of fluid overload, acidosis, and electrolyte imbalance, lower cost, and good survival. These results need confirmation in a wider study comparing RRT modalities in such children.
